# Analytical Techniques for the Characterization and Quantification of Monoclonal Antibodies

**DOI:** 10.3390/ph16020291

**Published:** 2023-02-14

**Authors:** Hassan A. Alhazmi, Mohammed Albratty

**Affiliations:** 1 Department of Pharmaceutical Chemistry and Pharmacognosy, College of Pharmacy, Jazan University, Jazan 45142, Saudi Arabia; 2 Substance Abuse and Toxicology Research Centre, Jazan University, Jazan 45142, Saudi Arabia

**Keywords:** analytical techniques, chromatographic, electrochemical, electrophoretic, monoclonal antibodies, spectroscopic

## Abstract

Monoclonal antibodies (mAbs) are a fast-growing class of biopharmaceuticals. They are widely used in the identification and detection of cell makers, serum analytes, and pathogenic agents, and are remarkably used for the cure of autoimmune diseases, infectious diseases, or malignancies. The successful application of therapeutic mAbs is based on their ability to precisely interact with their appropriate target sites. The precision of mAbs rely on the isolation techniques delivering pure, consistent, stable, and safe lots that can be used for analytical, diagnostic, or therapeutic applications. During the creation of a biologic, the key quality features of a particular mAb, such as structure, post-translational modifications, and activities at the biomolecular and cellular levels, must be characterized and profiled in great detail. This implies the requirement of powerful state of the art analytical techniques for quality control and characterization of mAbs. Until now, various analytical techniques have been developed to characterize and quantify the mAbs according to the regulatory guidelines. The present review summarizes the major techniques used for the analyses of mAbs which include chromatographic, electrophoretic, spectroscopic, and electrochemical methods in addition to the modifications in these methods for improving the quality of mAbs. This compilation of major analytical techniques will help students and researchers to have an overview of the methodologies employed by the biopharmaceutical industry for structural characterization of mAbs for eventual release of therapeutics in the drug market.

## 1. Introduction

Monoclonal antibodies (mAbs) are a mixture of analogous antibody molecules having monovalent affinity towards a defined antigen. These are synthesized via hybridoma technology that allows the production of mAbs at large scale with increased purity. Hybridoma technique involves the fusion of normal B-cell (desired antibody-producing splenocytes) to myeloma cell (immortal, cancerous B cells), ultimately generating a pool of single cell type secreting the identical antibody. A selection media, hypoxanthine aminopterin thymidine (HAT), is then used where only hybridoma cells can grow and further screened for the desired mAb. Orthoclone OKT3 (muromonab-CD3) was the first licensed monoclonal antibody, released in 1986 to prevent kidney transplant rejection [1]. The mAbs bind only to a particular epitope on the antigen which contrasts with polyclonal antibodies that bind to many epitopes on an antigen [2]. This makes mAbs functionally advantageous over polyclonal antibodies in terms of specificity and reproducibility. On the other hand, recombinant antibodies (rAbs) have also emerged which are in vitro generated mAbs from genes expressed in high efficiency expression vectors. In contrast to mAbs that are produced using conventional hybridoma-based technologies, rAbs do not require hybridomas and animals in their production [3]. The mAbs are widely employed in fields like research and diagnostics; therapeutic solutions for cancers and immunological disorders; and pharmaceuticals resulting in high market demand [4]. The human trials of mAbs have shown their immensely improved biological compatibility and reduced adverse effects (immunogenicity) [5]. Successful experimental trials of mAbs have extended their use from immune disorders and oncology to other ailments like migraine, infectious, and genetic disorders.

Besides the therapeutic use of mAbs, they can be used for diagnostic purposes (biochemical analysis, diagnostic imaging) and protein purification. The mAbs are very potent biological agents to evaluate various diagnostic assays, which include immunohistochemistry (IHC), enzyme-linked immunosorbent assay (ELISA), western immunoblotting, immunofluorscent antibody test (IFAT), flow cytometry, and radioimmuno assay (RIA) [5,6]. The mAbs are also used for molecular imaging in various pathologies, such as oncology, autoimmune diseases, and cardiovascular diseases, where mAbs target the imaging agents to the diseased sites in vivo. Moreover, mAbs are used for protein purification through the immunoaffinity chromatography (IAC) technique where the stationary phase comprises of mAbs as they have distinctive specificity for the desired protein, ultimately minimizing the contamination by unwanted molecules [6]. Several glutamylated polypeptides were identified by using mAb GT335 (glutamylated tubulin). At present, nearly 80 mAbs have been approved by regulatory agencies like the United States Food and Drug Administration (USFDA) and European Medicines Agency (EMA) [5,6]. Consequently, a rise of 7.1% in the compound annual growth rate (CAGR) of the mAbs global market is expected since the year, 2020. The demand for analytical methodologies optimized for rigorous characterization of mAbs has grown as the number of certified mAbs in the pharmaceutical industry has expanded with simultaneous entry of the potent biosimilars hitting the market. In this work we have attempted to give an overview of major analytical techniques for mAb characterization which will be useful to students, researchers, and personnel from the biopharmaceutical industry.

## 2. Structure of mAbs

**Figure 1:**
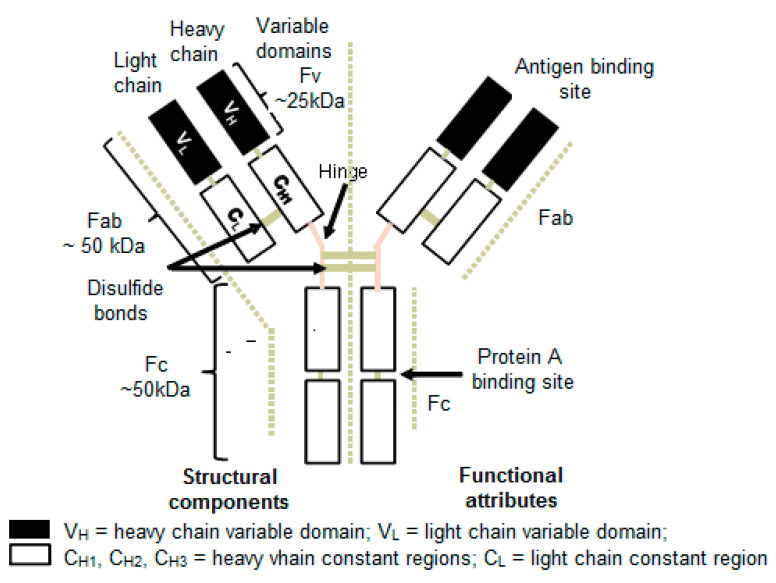
Structure of a Monoclonal Antibody (IgG).

Monoclonal antibodies are basically glycoproteins of the Ig (immunoglobulin) superfamily, and its five isotypes are categorized as: IgA, IgD, IgE, IgG and IgM. Among these isotypes, the IgGs are frequently used for therapeutic applications. The IgGs are high molecular weight (~150 kDa) complex glycoproteins and comprise of two identical light and heavy chains of molecular weight of ~25 kDa and ~50 kDa, respectively, that are joined by disulfide bonds and non-covalent bonding at their pivotal point (Figure 1). In this way, the formed tetramer creates Y-like shapes with two identical halves. Constant and variable domains are formed by the intra-chain disulfide bonds in the polypeptide chains [7]. The antibody can be divided into two main regions: the Fab (antigen binding fragment) that recognizes the antigen; and the Fc (crystallizable fragment) that interacts with other elements of the immune system, such as phagocytes to promote removal of the antigen. The Fab consists of two variable and two constant domains. The variable domain known as variable fragment (Fv) provides specificity for the antigen whereas the constant domain acts as a structural framework. Each variable domain consists of three hypervariable loops, known as complementarity determining regions (CDRs), evenly distributed between four less variable framework (FR) regions.

**Figure 2:**
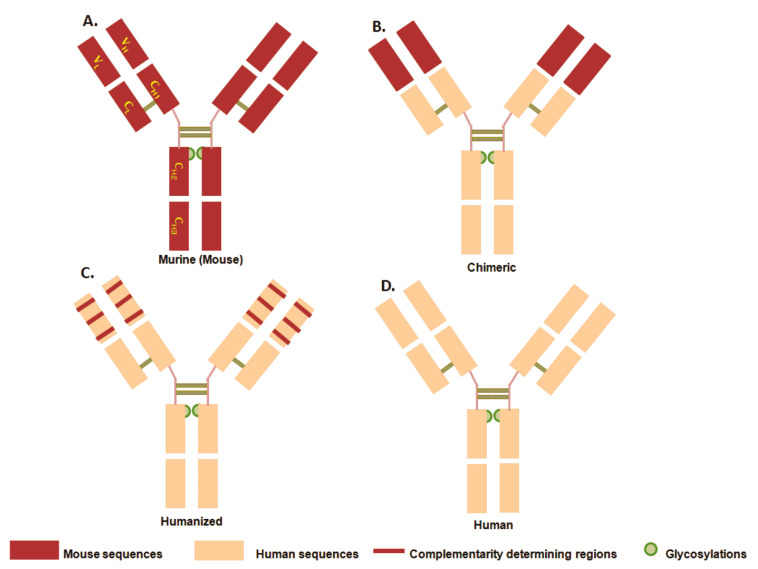
Four generations of Monoclonal Antibodies (mAbs); (**A**): murine; (**B**): Chimeric; (**C**): Humanized and (**D**): Human.

The CDRs provide the specific antigen recognition site on the surface of the antibody and the hyper-variability in this region enables the antibody to recognize different antigens. Therapeutic mAbs came into existence in four generations. First generation: murine antibodies (-momab) that were developed from mice and had 100% mouse protein but with the limitation of high immunogenicity. Second generation: chimeric antibodies (-ximab) with 33% mouse protein that contained variable domains of murine mAbs and constant domain of human antibody and showed lower immunogenicity. Third generation: humanized antibody (-zumab) generated by techniques, such as CDR (complementarity determining regions) and SDR (specificity determining residues) grafting to increase the human content >90% in the antibody. This is done by grafting CDRs onto the backbone of human Ig, both constant regions and the frames of the variable regions. Fourth generation: human antibodies (-umab) having 100% human content in the antibody and very low immunogenecity (Figure 2) [8,9,10]. However, the mAbs are produced through hybridoma technology; it involves several post-translational modifications (PTMs), glucosylation, and methionine oxidation, leading to the formation of antibody-charge size variants on the peptide chains during manufacturing, storage, and post-administration in vivo or during clinical trials [11,12]. The PTMs account for the additional heterogeneity (macro- or micro-variations) and complexity to the protein. Most micro-heterogeneities contribute to variability in the pharmacological attributes of mAbs, such as half-life, antigen binding, anti-inflammatory action, or elevated immunogenic responses and, therefore, are considered as critical quality attributes (CQAs) of mAbs [13,14]. The high complexity of mAbs poses a great analytical challenge and in-depth characterization of mAbs is a necessary requirement at all stages of development. 

## 3. Characterization of mAbs

During a biopharmaceutical product development, thorough characterization must be performed according to the regulatory guidelines, such as the EMA and documented in “Guideline on development, production, characterization and specification for monoclonal antibodies and related products” (July 2016) [15]. The detailed characterization involves elucidation of structural, physiochemical, immunological, biological properties on one hand; and assessment of impurities and quantification of antibody on the other hand prior to clinical trials and eventual commercial release as a therapeutic drug. Table 1 illustrates the different aspects of antibody characterization and the most common analytical method(s) in this respect. 

### 3.1. Structural and Physiochemical Characterization

The structural and physiochemical characterization of biopharmaceutical products as stated by harmonized ICH guideline Q6B [16] should encompass amino acid sequence analyses, N-terminal and C-terminal sequencing, peptide mapping, identification of free sulfhydryl groups and disulfide bridges, carbohydrate content, glycosylation, glycan structure, and post translational modifications, such as oxidation, deamidation etc. The mAbs normally consist of one N-glycosylation site on each heavy chain located in the Fc region and their presence or absence must be verified in the mAbs. The secondary, tertiary, and quaternary structures are collectively known as higher order structures (HOS) and are responsible for the 3-D shape and correct folding of the biopharmaceutical [17]. Incorrect 3D shape affects protein functionality and may lead to inhibition of antigen binding, exposure of immunogenic epitopes and aggregation of protein. Different analytical techniques (Instrumentations) employed for the characterization of mAbs are described in Table 1.

### 3.2. Immunological Properties

The binding assays of the antibody to antigen and identification of complementary determining regions (CDR) must be analyzed. Moreover, the epitope and the molecule bearing their relevant epitope have also been determined through immuno-histochemical procedures [17]. The determinations of affinity, avidity, and immunoreactivity have been accomplished through Enzyme Linked Immuno-Sorbent Assays (ELISA) and Surface Plasmon Resonance (SPR) [18] (Table 1). Both ELISA and SPR are considered as complementary techniques and provide consistent results as far as the characterization of mAbs is concerned. These techniques can also be used to study the mAb-antigen complex and provide the affinity values in form of equilibrium dissociation constant. The biological activities as well as the pharmacokinetics of mAbs depend intrinsically on their binding to the target antigen and controlling this binding is important for the characterization of mAbs [18]. The SPR technology offers great application in achieving the characterization of mAbs as it can measure binding to receptors, binding to antigens, along with the measurement of the active concentration required for binding. The SPR technique helps in determining the epitope specificity which is an important part of the mAb characterization. 

### 3.3. Biological Activities

The biological activities must be evaluated through suitable in vitro (enzymatic/radioisotope/florescence methods) and in vivo assays. A detailed analysis of antibody-dependent cellular cytotoxicity (ADCC), cytotoxic properties (apoptosis), the ability for complement binding, and activation and neonatal Fc receptor binding activity, has also been studied to identify the mechanism of action and importance of the product effector functions [19]. The mechanism behind the biological activity of mAbs include immune-mediated cell toxicity, direct cell toxicity, immune system modulation, as well as disruption of the vascular system. Owing to various advancements in the development of mAbs, they can be utilized in the treatment of various cancers and autoimmune disorders with an added advantage of reduced adverse effects as observed with traditional treatment strategies. However, considerable research is yet to be made to identify novel targets for mAbs and to improve its drug ability. The mAbs constitute a part of the multi-disciplinary treatment approach adopted for cancer patients and has proven to be greatly beneficial in enhancing the quality-of-life of cancer patients. 

### 3.4. Purity and Contaminants

Generally, mAbs display various heterogeneity resources, such as C-terminal lysine processing, oxidation, deamidation, fragmentation, isomerization, disulfide bond mismatch, glycation, N-linked oligosaccharide), which lead to a complex purity/impurity profile comprising several molecular entities or variants [20]. These include determination of physicochemical properties, for example, molecular weight or size, isoform pattern, extinction coefficient, electrophoretic profiles (based on size and charge), chromatographic data (based on size, charge, hydrophobicity/hydrophilicity), and spectroscopic profiles (structure analysis, such as secondary and tertiary). Furthermore, contaminants, such as microbial species and endotoxins, must be prohibited properly, or completely removed. On the other side, the application of additional analysis, e.g., a monocyte activation test, have been attempted to eradicate the presence of non-endotoxin pro-inflammatory contaminants (peptidoglycan) [21].

### 3.5. Quantification

The physicochemical and immunochemical assay have been utilized for the determination of the quantity of mAbs. The observed quantity values demonstrated direct correlation with the biological assay [22]. The instrumentation, e.g., colorimetric assays, HPLC or ion chromatography (IC), have been reported earlier for quantification of the mAbs (Table 1).

**Table 1:** Methodologies and instrumentation for characterization of monoclonal antibodies.

Type of Characterization		Details of Methodologies	Analytical Methods/Instrumentation	References
Structural	sequence of amino acids	Edman chemistry, Mass-spectrometric sequencing	enzymatic or chemical digestion with LC-MS/MS; ESI-MS analysis and/or MALDI-TOF MS	[23]
	composition of amino acids	amino acid analysis	Amino acid Analyzer	[24]
	amino-acid sequences at N- and C-terminals	identification of free amino acid or a pyroglutamic acid in the N-terminal region; identification of lysine(s) in the C-terminal on the heavy chain	LC-MS/MS	[25]
	peptide map	peptide mapping	Online LC-MS (with MS/MS and/or MSe)	[26]
	free sulphydryl groups and disulfide bridges	identification of expected and mismatched disulphide bridges	LC-MS/MS; peptide mapping; MALDI-TOF; Electrospray MS; colorimetric tests	[27]
	carbohydrate content	structure of the carbohydrate chains; amino sugars, neutral sugars and sialic acids; the oligosaccharide pattern (antennary profile)	selective enzymatic cleavage and MALDI-TOF MS; HPLC; HILIC; CE-LIF or IEX	[28]
	glycan structures	level of galactosylation, mannosylation, sialylation and fucosylation; presence and distribution of main glycan structures	peptide mapping; GC-MS; enzyme array; CE or normal phase LC; MALDI-TOF	[29]
	Post-translational modifications	deamidation, glycosaylation, oxidation, phosphorylation, alkylation, acetylation, methylation, sulfation, truncations etc.	MS (Orbitrap and QToF)	[30]
	higher-order structure (HOS)	secondary and tertiary structural features (á-helix, â-sheet, â-turns and random coil/unfolded)	Circular Dichroism (CD)	[31]
		secondary structure estimation (deconvolution of the amide-1 band);	Fourier transform-infra red spectroscopy (FT-IR)	[32]
		3D structure determination	Nuclear magnetic resonance spectroscopy (NMR): 1D and 2D NOESY and TOCSY	[33]
		qualitative tertiary structural information	Intrinsic fluorescence spectroscopy; UV-vis spectroscopy	[34]
	aggregation studies	presence of irreversible protein oligomers and higher order aggregates	Size exclusion chromatography (SEC); Sedimentation velocity analytical ultracentrifugation (SV-AUC); Dynamic light scattering (DLS)	[35]
Immunological	binding assays of the antibody to antigen; identification of complementary determining regions (CDR)	Determination of affinity, avidity and immuno-reactivity	Enzyme-Linked Immunosorbent Assays (ELISA); Surface Plasmon Resonance (SPR)	[36,37]
Biological	Cytotoxic properties such as apoptosis, competent binding ability, activation and other effector functions	Antibody-dependent cellular cytotoxicity (ADCC), anti-proliferation, migration etc.	In vitro cell-based bioassays by enzymatic/radioisotope/florescence methods	[38]
Purity and contaminants	Estimation of physico-chemical properties	molecular weight/size	Orbitrap, QToF; MALDI; size exclusion chromatography (SEC); reducing and/or non-reducing SDS-PGE	[39]
		isoform pattern	Chromatography and traditional gel/capillary electrophoresis: IEX, CIEF, CZE and SDS-PAGE; Imaged capillary isoelectric focusing (icIEF)	[40]
		extinction coefficient	Amino Acid Analyzer (AAA) or UPLC	[41]
	electrophoretic profiles	electrophoretic analysis based on size and charge	SDS-polyacrylamide gel electrophoresis, western blot/capillary electrophoresis; capillary isoelectric focusing (CIEF/iCIEF); polyacrylamide gel electrophoresis	[42]
	chromatographic profiles	chromatographic analysis based on size, charge, hydrophobicity/hydrophilicity	Reversed-phase liquid chromatography (RP-HPLC), size exclusion chromatography (SEC), ion exchange chromatography (IEX)	[43]
	spectroscopic profiles	structure analysis (secondary and tertiary)	Ultraviolet-visible (UV-Vis) spectroscopy, intrinsic fluorescence studies, near and far-UV circular dichroism, Nuclear magnetic resonance (NMR), Fourier transform-infrared infrared (FT-IR) spectroscopy	[44]
	multimers and aggregates	Field Flow Fractionation (FFF), size-based chromatography, and Analytical Ultra Centrifugation (AUC)	SEC with multi-angle laser light scattering (SEC-MALS), sedimentation velocity analytical ultracentrifugation (SV-AUC) and dynamic light scattering (DLS)	[45]
	process and product related impurities; contaminants	presence of host cell protein, DNA, cell culture and other residues; microbial species, endotoxins	GC-MS, LC-MS/MS; real time multiplex PCR based assays	[46]
Quantification		estimation of total amount of mAB	colorimetric assays; HPLC or ion chromatography (IC)	[47]

Assessment of the binding of the antibody to the purified antigen in customized binding assays determines the immunological properties of the developed monoclonal antibody. The functional assessment of the antibody can be achieved by various in vitro cell-based assays which shed light on cytotoxic properties and effector functions of the antibody [48]. The quantity of the antibody is one of the important parameters determined by various calorimetric assays or by chromatography methods. In addition to the above criteria, production process and product related impurities and contaminants must be identified and quantitatively and qualitatively evaluated. Therefore, there is an important and urgent need for writing a comprehensive review on the state of the art of the useful analytical techniques for quality control of mAbs to meet the acceptable standards of regulatory guidelines. In general, each new batch of mAbs is essentially required to be tested for the identity, heterogeneity, impurity content, and activity before release. The most common techniques for various mAb analyses are listed in Table 2.

## 4. Analytical Techniques for mAbs Analyses

New and ever-evolving analysis strategies have been developed for monoclonal antibody characterization which involve chromatographic, electrophoresis, spectroscopic, electrochemical, and other methods. Among these techniques, capillary electrophoresis (CE) has gained significant interest because of its high resolving power and effectiveness in separating mAbs and its analogues [97,98]. Capillary electrophoresis is used to characterize the mAbs and its derivatives both physically and chemically covering the site-specific characterization, peptide mapping, heterogeneity assessment based on charge and size, glycosylation profiling, impurity analysis, stability determination, and biosimilarity assessment [97]. Several electrophoretic approaches, such as capillary gel electrophoresis (CGE), capillary isoelectric focusing (cIEF), and capillary zone electrophoresis (CZE), are frequently used for the analysis of mAbs. Several putative post-translational modifications (PTMs) can alter the charge distribution of mAbs which, may significantly affect its biological property. Therefore, ion-exchange chromatography (IEX) has also been accepted as the standard mode for the characterization of mAb charge variants, which are considered as important quality parameters for stability and process consistency [99]. On the other hand, 1D and 2D Nuclear Magnetic Resonance (NMR) has been utilized by several researchers to obtain highly specific High Ordered Structures (HOS) of mAbs. Two- dimensional NMR can indicate molecular fingerprints of proteins at atomic resolution level providing detailed structural information [49]. These analytical processes facilitate the separation of the required mAbs isoform and deliver a thorough characterization, illustrating the organization of the protein. The following sections describe major analytical techniques and certain modifications applied in the existing protocols for the analyses of mAbs.

### 4.1. Chromatographic Methods

The chromatographic method allows separation of the components of a mixture between two phases based on their physico-chemical properties. The PTMs in proteins including glycosylation, nitrosylation, lipidation, phosphorylation, acetylation, ubiquitination, and methylation, can modify proteins resulting in different variants that can be easily separated [100].

#### 4.1.1. Reversed-Phase Liquid Chromatography (RPLC)

Despite that IgG antibodies are highly stable molecules, they are nonetheless susceptible to post translational modifications [101] and several degradation reactions can occur during different stages of synthesis, formulation and storage [102,103,104]. Even in a minimal form and in low quantities, these variations can generate significant structural and biological changes in mAbs, ultimately leading to diminished bioactivity; therefore, it becomes imperative to identify and quantify the variations existing in the recombinant monoclonal antibodies (mAbs) before proceeding for biopharmaceutical development. The RPLC is one of the common techniques which has very good resolution in evaluating protein variations arising from different chemical reactions or post-translational modifications. Depending upon the discrete examination of mAb subdomains, Yan et al. [105] revealed a novel method for assessing variants in mAbs. Reduction by dithiothreitol and papain cleavage was used to create these subdomains. A reversed-phase LC-MS (RPLC-MS) methodology was applied to separate antibody subdomains (light and heavy chains, Fab and Fc) with numerous specific alterations including pyroglutamic acid, isomerization, deamidation, and oxidation. This report suggested the way for analyzing antibody variations using multimodal methods and assessing the mAb heterogeneity both qualitatively and quantitatively [105]. The RPLC is a critical subtype of high-performance liquid chromatography (HPLC) and is a variant of Bonded Phase Chromatography. In contrast to normal or linear phase systems, this technique inverts the polarity of the original adsorbent in addition to the mobile phase polarity when matched against the original Normal Phase. The extensive use and popularity of RPLC can be attributed to its flexibility and adaptability with a versatility of separating practically every kind of analyte [106].

Fekete et al. [50] proposed and functionally demonstrated a new 3D retention model for large therapeutic protein like IgG_1_ cysteine conjugated ADC (antibody drug conjugate) in RPLC with an aim to quantify an ADC product’s average drug to antibody ratio (DAR). A rapid and effective resolution of the DAR species of a commercialized ADC sample, notably brentuximab vedotin, was obtained in some preliminary studies. It was discovered that a generic (platform) methodology development approach was feasible. When compared to the trial-and-error approach, this methodical process saved time. Finally, based on the observed peak area ratios of the RPLC chromatogram of the decreased ADC sample, RPLC can be deemed a good approach for determining the average DAR of an ADC [50]. To boost monoclonal antibody-based treatments, researchers are now concentrating their efforts on converting these biomacromolecules into nanoparticles. According to the International Conference on Harmonization (ICH) recommendations, Sousa et al. [51] tested and applied RPLC with fluorescence detection method for identification of bevacizumab (potent human mAb used in treating various malignancies and optical disorders) from nanoparticulate systems. For the first time, this technology was utilized to determine and correlate the quantity of bevacizumab encased in poly(lactic-co-glycolic acid)-based nanoparticles pre and post lyophilization.

Therapeutic mAbs are frequently analyzed using RPLC at high temperatures (80–90 °C) accompanied with high TFA concentrations (0.1%). It is possible to attain appropriate performance based on peak shapes and recoveries under such settings. A wide-pore silica-based superficially porous (SPP) material treated with phenyl bonding was lately presented by Bobaly et al. [52], which offers a way to execute protein RPLC under more benign conditions in terms of temperatures and TFA concentrations. Twenty-three mAbs approved by USFDA and EMA were tested on this novel column, in addition to the standard C4 SPP widepore silica-based column, to assess the feasibility. This study showed that even moderate conditions (65 °C and 0.03% TFA + 0.07% FA) may be used to successfully analyze mAb subunits using RPLC-MS. Appropriate stationary phase with milder conditions was used to enhance MS sensitivity while keeping elution profiles comparable. Furthermore, using milder settings may lower the likelihood of artifact peaks. Multi-angle light scattering (MALS) coupled to RP-UPLC (Reverse phase-Ultra high-pressure liquid chromatography) is a traditional method for characterizing intact mAbs and their fragments using low-dispersion MALS detector. In RP gradients, Gentiluomo et al. [53] validated a constant refractive index growth value for mAbs and found these to be consistent to those earlier reported for other protein classes. In fact, proteins with low thermal and physical stability can combine under the severe conditions used in RP separation of mAbs (i.e., organic solvents at high temperatures). In addition, they used RP-UPLC-MALS in long-term stability investigations. The RP-UPLC-MALS method uses a new separation principle, which adds another level of protein characterization by calculating the molecular weight for each individual peak of resultant chromatogram allowing the identification of desired monomeric chemical variants and other impurities or degradation products (aggregates and fragments).

For comprehensive characterization of mAbs, such as deducing the sequence of amino-acids and identification of post-translational modifications and degradations, peptide mapping is the preferred approach which necessitates full peptides separation and absolute sequence coverage [107]. However, generally, RPLC-MS does not successfully offer complete coverage as small peptides (that are hydrophilic in nature) are lost in the void volume and during strong column interactions with larger hydrophobic peptides [107]. 

#### 4.1.2. Size-Exclusion Chromatography (SEC)

Size exclusion chromatography (SEC) is another important method for the separation and purification of mAbs from the other heterogenic impurities which may cause immunogenic responses [108]. These impurities include components that are smaller and larger than the intact mAb; where smaller components are produced via enzymic or non-enzymic cleavage and the partial creation of uneven disulfide linkages. On the other hand, larger components result from molecular aggregation, association, or even through precipitation [109]. The biopharmaceutical industry uses SEC to monitor the purity level of mAbs for the quality control (QC) process, as more than 99% purity is required for their medicinal use [108]. The SEC used to separate the molecules depending on the molecule size leading to the elution of larger molecules first from the column. Depending on the molecular size differences, biomolecules spread through the stationary phase comprising of porous spherical particles whereby an aqueous buffer is used as the mobile phase [110]. The SEC columns are polymer or silica-based; however, currently covalently modified diol hydrophilic layer is used in the columns that reduces imprecise proteins binding to the silica. The conditions of flow rate, mass load, volume load and column length can be evaluated in chromatography and the modification of these factors might impact resolution, time duration of analysis and precision [110]. Separation ability of an SEC column is directly proportional to the square root of the column length; hence several columns in succession can be connected in situations that require separation on long columns [111].

Several developments have been reported in SEC to improve the output and resolution which include the use of smaller and thinner columns packed with minute-sized particle (below 3 µm), however, this increases the possibility of degradation due to high pressure [109]. Multiplexing of several detectors may be applied in SEC including UV (ultraviolet), RI (refractive index), IV (viscometer), and MALLS (multi-angle laser light scattering), for extensive characterization of protein samples [111]. Mixed mode SEC chromatography protocol was generated for the analysis of mAb oxidation variants followed by SEC-MALLS for pre-peak monomers characterization and confirmation by LC–MS [54]. The development of size exclusion–ultra high-pressure liquid chromatography (SE-UHPLC) is a major progress in the area of SEC in which SE-UHPLC uses instrumentation containing decreased system volume and higher acceptable back pressures compared to typical HPLC instruments [55]. The SE-UHPLC has been confirmed for high-throughput, high-resolution separations of polymers and proteins [112] and has been validated for practice in QC laboratories [113].

The SEC analysis of more complex novel biotherapeutic products, especially, bispecific mAbs, hydrophobic molecules, Antibody Drug Conjugates (ADCs), and co-formulations, requires unique considerations during the development of SEC methods and execution [114]. In the case of hydrophobic molecules and ADCs, supplementation of organic substitute in the mobile phase, disrupts the hydrophobic bonding amid protein and stationary phases that results in the reduced elution time of the main peak [115]. Generally, more size variants are present in bispecific molecules and co-formulations as compared to standard mAbs, e.g., bispecific antibodies have undesirable product variants like homodimer, miss-paired or scrambled light chain, and single arm half-antibodies. For the quick characterization of bispecific molecules, SEC is directly attached to SEC–MS [116]. A better resolution can be achieved when SEC is used with SE-UHPLC compared to SE-HPLC [113]. For analysis of co-formulated or co-mixed biotherapeutics, a classic SEC method, such as the USP <129> SE-HPLC method, can be used [117], however it may not be suitable for two drugs with similar molecular weights. Hence, to improve the further separation, SE-UHPLC or multiple SE-UHPLC columns in series can be used [109]. Furthermore, with above mentioned strategies, other techniques for attaining the improved peak separation may be developed, for example multidimensional liquid chromatography [118]. Proteins (like-antibodies) with very comparable sizes cannot be separated by SEC, however they can be distinguished by IEX chromatography if their isoelectric point (pI) values are different. In IEX chromatography, antibodies emerge as two peaks owing to variations as acidic and basic species [119].

#### 4.1.3. Ion-Exchange Chromatography (IEX)

Ion-exchange chromatography (IEX) is an established and most popular method employed to purify and characterize curative proteins based on the use of electrostatic connection amid the protein sample and resin. Ion-exchange chromatography is broadly classified into cation-exchange (CEX) or anion-exchange (AEX) chromatography; however, CEX is more commonly used for protein characterization [56]. The most typical application of IEX chromatography is to separate target proteins from complexes, host cell proteins, and other impurities as an interim purification step. Through IEX, protein in different oligomeric states [120] in addition to protein isoforms [121] and proteins covalently linked to other biomolecules (like glycoproteins) [122,123] could easily be separated. Conventional pH and linear salt gradient could easily be applied to achieve separation in IEX [123,124,125]. In contrast to SEC, many conditions, such as the gradient slope, concentration and composition of salts, pH of buffer, and type of matrix and ligand, could be modified [126]. In traditional IEX, a constant linear salt gradient is provided for elution, and proteins are eluted in ascending order of binding charge. For this, pH of the buffer needs to be adjusted according to the pI value of every antibody to be eluted. A committed pH gradient buffer system can be developed to minimize time, consumption for samples with a broad range of pIs. The variants of most compounds exhibit improved resolution through this salt gradient method in addition to the higher peak capacities establishing the superiority of salt gradient CEX over the pH gradient CEX for antibody analyses [56].

Ishihara and Yamamoto [127] used simple methods for the optimization of IEX and suggested that protein adsorption relies on several factors, such as the protein structure and sample yield, operating parameters, e.g., buffer pH and constitution, sample volume loading, flow rate, and the physical characteristics of the adsorbent matrix. Further improvements in IEX, include the use of a novel cation-exchange resin, Eshmuno™ S, as an optional antibody trapping step combining high volumes, yields and the capacity of considerably reducing host cell contamination resulting into high flow rates, and consequently high performance [128]. Another advancement to characterize the biomolecules combined natural charge variant partitioning that incorporates high resolving native mass spectrometry [57]. Recently, new interventions on the interactions between the column, protein, and buffer ingredients have been reported. The report corroborates some common misconceptions concerning ion-exchange elution mechanisms and makes recommendations for method improvement [58]. 

### 4.2. Electrophoretic Methods

Capillary electrophoresis (CE) is one of the important approaches for analyzing biological therapeutic molecules because of the unique feature of separation with a high resolution in miniature configuration [98]. Capillary electrophoresis acquires attributes that are complementary to and different from liquid chromatography [59]; itis a significant approach for analyzing biotherapeutic compounds based on electromigration in aqueous buffers, preserving the high-order structure of biomolecules [129,130,131]. Coupled with MS, CE further improves biomolecule identification by separating analytes that migrate at different mass-to-charge ratios [131]. Capillary electrophoresis modes, such as capillary zone electrophoresis (CZE), capillary isoelectric focusing (cIEF), and capillary gel electrophoresis (CGE), have been innovated for profiling of mAbs at various levels [98] which are discussed here.

#### 4.2.1. Capillary Zone Electrophoresis (CZE)

Capillary zone electrophoresis (CZE) has been recognized as free solution capillary electrophoresis. It is the most basic kind of capillary electrophoresis. The mechanism of separation is based on the charge-to-mass ratio disparities. The CZE’s principles include consistent field strength with homogeneity of the buffer solution over the entire length of the capillary [132]. For mAbs analysis, CZE is a versatile, cost-effective, and extensively used CE technique. Its charge-to-hydrodynamic-ratio-based separation method allows it to analyze mAbs at many levels with high resolution and efficiency [59]. Due to the lack of a stationary phase, it is modified at the level of sample preparation (in-line pre-concentration, in-line digestion, enzymes, aqueous solvent, temperature control) and electrophoretic separation (denaturing or native conditions) [97]. One major issue during characterization of mAbs and ADCs by CZE is the adsorption of analytes on the capillary wall, which alters peak efficiencies and analysis reliability that leads to mAb loss [133]. To avoid adsorption, electro-osmotic flow (EOF) is adjusted which increases analysis reproducibility. Furthermore, a coating is usually added to the background electrolyte (BGE), which gets adsorbed reversibly to the capillary wall [134]. Dynamic coating with polymers like cellulose derivatives: hydroxypropyl methylcellulose (HPMC) and hydroxypropyl cellulose (HPC); polyamines including triethylenetetramine (TETA), or polyethylene oxide (PEO) are mentioned in several techniques in the literature [60,135,136]. The active coatings, on the other hand, create strong noise signals, ion source contaminants, and ion repression when CZE is coupled with MS [134]. Because they are absorbed permanently or covalently to the capillary wall, permanent coatings are superior for high stability. Inert coatings like linear polyacrylamide (LPA), polyvinyl alcohol, and positively charged coats like cross-linked polyethyleneimine and 1-(4-iodobutyl)4-aza-1-azoniabicyclo[2,2,2]octane iodide (M7C4I) are often utilized for mAb characterization [61,62]. In addition, some researchers have used efficient rinsing protocol to remove the adsorbed ions on the inner capillary wall and regenerate the capillary in CE analysis [130,137].

The charge heterogeneity in antibodies was studied using a high-throughput microchip-CZE approach [138]. Derivatization of protein molecules with Cy5 N-hydroxysuccinimide ester (NHS-ester) allows fluorescence detection of protein on commercial microchip equipment without affecting the charge profile of the protein [138]. The ZipChip microfluidic CE-ESI-MS device was utilized to successfully identify proteoforms of the medicinal products rituximAb, trastuzumAb, and bevacizumAb [63]. To facilitate structural characterization, CZE is usually combined with MS and/or an on-line optical detector (UV, LIF). To characterize mAbs, ESI-MS with sheath liquid or sheathless interfaces are often used [59]. The sheathless interface is connected to a conductive liquid reservoir via a porous tip at the capillary output. It has a low flow rate and no dilution effect, and because of its better sensitivity and selectivity, it is used more commonly for mAbs characterization than previous interfaces [107,139]. Design of experiments (DoE) models were created and enhanced at multiple levels for CZE technique optimization, including resolution, peak width, and number of peaks. Furthermore, pH, identification of the polymer additive, and the quantities of additives, such as butanolamine, acetonitrile, and TETA, can all help to minimize optimization efforts [64].

#### 4.2.2. Capillary Gel Electrophoresis (CGE)

Capillary gel electrophoresis (CGE) is a modified version of CZE that uses gel instead of liquid within the tube to separate species by size. Capillary gel electrophoresis is a conventional method for analyzing mAbs since the 1990s. It aids in determining the apparent molecular weight of molecules and allows for the evaluation of product size heterogeneity, stability, and purity [140,141,142,143]. For CGE, the sample is heated with a high concentration of SDS, which denatures the secondary and tertiary structures without disturbing the disulfide linkages, resulting in proteins that are evenly charged. The capillary tube is packed with a polymer-based sieve matrix, which causes electrophoretic separation based on the hydrodynamic radius of the proteins [144]. The non-reduced and reduced conditions are used to examine mAb size heterogeneity. Reduced CE-SDS is extensively used to study fragments of mAbs, whereas non-reduced CE-SDS is generally employed to control aggregation/purity [98]. Xie et al. [145] used the non-reduced CE-SDS approach to measure the degree of reduced disulfide bonds in mAbs [145]. In a separate study, non-reducing and reducing CE-SDS were employed to investigate the U.S. Pharmacopoeia IgG standard’s system suitability acceptance criterion [146].

The CGE analysis is traditionally accomplished by combining UV or fluorescence detection. Mostly, UV detection is programmed at wavelength 220 nm and sometimes 200 nm, 214 nm, and 280 nm [98]. However, when compared to UV detection, laser-induced fluorescence (LIF) detection has a better sensitivity [98]. Fluorescence excitation is typically set at 488 nm for CGE-LIF analysis, and the emission signal is examined at 520 or 560 nm [97]. Preferred method for the mAb glycosylations analysis is the chemical alteration relying on 8-aminopyrene-1,3,6-trisulfonic-acid (APTS) (excitation: 488 nm, emission: 520 nm) reaction permitting rapid electrophoretic separation, with a greater effectiveness and a higher sensitivity [147,148]. However, other dye reagents, such as Teal™ (excitation: 488 nm and emission: 520 nm) [149] and 2-aminobenzoic acid (2-AA) (excitation: 325 nm emission: 405 nm) [150], have also been used for the glycan assay. Investigations on mAb fragmentation provide significant knowledge about the mechanisms of mAb degradation which can help to enhance mAb production and storage [151,152,153].

Despite that MS and CGE-SDS are incompatible because sieving matrix components cause substantial ion suppression, numerous attempts have been undertaken to reduce interference sources. Different in-capillary techniques for removing SDS from antibody samples treated with SDS, such as brief isotachophoresis, injections of organic solvents, cationic surfactants, or stripping agents, prior to MS detection (cyclodextrins) have been investigated [154]. Further, it has been shown that MS signal intensity can be increased above 94% using cetyl trimethyl ammonium bromide (CTAB) and methanol. Using a modified SDS-removal approach, a bi-dimensional CGE–SDS/CZE–MS system has been employed to distinguish different mAbs subunits and contaminants [153].

#### 4.2.3. Capillary-Isoelectric Focusing (cIEF)

Separation of species based on their isoelectric points (pIs) is the basic principle in capillary-isoelectric focusing (cIEF) analysis. In cIEF, a pH gradient is created inside the capillary tube and the targeted mAb travel under an electric field until their global charge is zero and the pH of sample becomes equal to its pI (isoelectric point)] [65]. Moreover, the solubility of mAb decreases when its pH reaches near to its pI and it likely gets precipitated. Hence, it becomes imperative to make an appropriate choice for the pH of the background electrolyte (BGE) as well as the sample buffer [154].

For analysis, a sample matrix containing a solution of mAb, carrier ampholytes, pI indicators and additives is filled into the capillary tube. Additives like surfactants and denaturants, aid in reducing the aggregation and precipitation of mAbs after focusing; the most common additive is urea. However, a shift in the apparent pI happens due to denaturing effect of urea [155,156]. The non-detergent taurine and sulfobetaine in the matrix formula are incorporated to provide good resolution and repeatability in imaging cIEF (imaged capillary isoelectric focusing/icIEF) analysis of mAbs performed in natural conditions without the addition of urea [157]. Moreover, the pH gradient, matrix viscosity, electro-osmotic flow (EOF), stability of mAbs, separation, resolution, peak morphologies, and migration duration, may all be affected by the salt presence in the mAbs sample matrix [158]. However, certain instrumentation remains difficult because of high concentrations and non-volatility of ampholytes and additives, as well as the diluting effect of the sheath liquid interface [159]. Apart from these issues, technical advancements in cIEF and icIEF detection techniques combined with MS have been made in recent years. For the characterization of charge variations, an online icIEF system with MS-compatible conditions has also been created [160].

In this way, an analysis by cIEF is influenced by a many parameters. As a result, some research used the design of experiments technique (DoE) to optimize analytic parameters. The DoE method was utilized to adjust the sample composition (ampholyte, urea, arginine, di-aminoacetic acid, HPMC, and pI markers) before being employed to analyze an ADC gemtuzumAb-ozogamicin [161]. Another study found that when using the DoE approach to optimize the cIEF method for NIST (National Institute of Standards and Technology) mAb analysis, the related sample preparation factors (concentration of carrier ampholytes, L-arginine, and urea) had a significant impact on separation, resolution, peak positions, and counts [162,163,164].

### 4.3. Spectroscopic Methods

To elucidate the specific high ordered structures (HOS) of mAbs, spectroscopic methods were also employed by different researchers during the last two decades [49,164]. Although several previously studied analytical methods, such as liquid chromatography, bioassays, diverse forms of electrophoresis, and peptide mapping have illustrated the covalent structure of the proteins (sequences of amino acid and post translational modifications), they all lacked the information about the HOS of mAbs [165]. In recent years, the application of numerous advanced spectroscopic strategies, for example circular dichroism (CD) and Raman and Fourier transform infrared spectroscopy (FTIR) were utilized to determine the tertiary structures of mAbs. These techniques involve specific conditions to perform the chemical analysis and reported the HOS of mAbs [49,66]. Consequently, NMR played a crucial role in elucidating the three-dimensional (3D) structure by providing the HOS of mAbs and is possibly the best method for the structural characterization of proteins especially the therapeutic mAbs. Recently, there has been a gradual augmentation in the use of therapeutic mAbs in the commercial market necessitating the implementation of a non-invasive detection method, such as NMR and other sophisticated techniques, to analyze tertiary structures of the biologics [67,166,167,168]. Different NMR techniques with emphasis on 1D ^1^H, 2D ^1^H-^15^N and ^1^H-^13^C NMR experiments have been used in the last five years for the characterization of HOS of mAbs.

#### 4.3.1. ^1^H-Based 1-Dimensional NMR

1-Dimensional NMR has been the most suitable method for determining the chemical shift and structural elucidation of the protein therapeutics. However, the requirements of isotope labeling, larger molecular weight and the restraints imposed due to numerous formulations have led to its decreased acceptability for mAb therapeutics. Therefore, to overcome this problem, PGSTE (pulsed field gradient stimulated echo) experiment was developed to characterize the formulated mAbs utilizing ^1^H NMR by generating highly resolved spectra of intact mAbs in their formulation buffers [164]. Additionally, the same group also demonstrated the PROFILE method (PROtein FIngerprint by Line shape Enhancement) to resolve the major drawbacks faced by applying the traditional 1D ^1^HNMR for fingerprinting of mAbs [164]. Elliot et al. [68] reported the latest alternative process for probing 1D diffusion edited spectra through employment of the combination of PROFOUND (PROtein Fingerprint Observed Using NIPALS Decomposition) and NIPALS (nonlinear iterative partial least squares) principal component analysis.

The reversible self-association of mAbs has also been reported to be monitored by 1D ^1^H NMR, which ultimately reveal the dimension of the transient protein clusters and viscosity of the solution [49,69,70]. In another study, the combined approach of fast protein liquid chromatography (FPLC)-NMR, CD, SEC, and MALS (multi-angle light scattering), was suitable for yielding an overall outline of HOS and different quinary structure of mAb therapeutics [71]. Recently, investigations of both the protein and stability of mAb formulations have been performed using 1D ^1^H NMR signal intensity through the protein degradation i.e., aggregation or fragmentation [72]. Furthermore, the combination of liquid-state NMR spectroscopy with cryo-electron microscopy and dynamic light scattering reported to play a critical role in drug delivery systems through characterizing various aspects of liposome-peptide formulation, such as peptide and liposome assembly, stability of the formulation, their mutual interactions, and the peptide release profiling [73].

#### 4.3.2. ^1^H-Based Multidimensional NMR

Multidimensional NMR spectra might individually resolve most of the sites in the spectrum and reveal a complete fingerprint of any protein. The demand of application of 1D and 2D NMR mainly 1D ^1^H, 2D ^1^H-^15^N and ^1^H-^13^C NMR have been escalating in recent years, mainly for the identification of quality attributes (QA) and HOS characterization of mAbs [169]. The QA and HOS have been reported to be very specific for the biopharmaceuticals and structural changes might lead to several drawbacks, which can be overcome by 2D-NMR methods by detecting the precise atomic-level fingerprint of the primary/secondary/tertiary and quaternary structures of therapeutic mAbs [169].

On the contrary, owing to the short transverse relaxation times along with the slow molecular tumbling, multidimensional NMR method has resulted in its lesser adaptability for structure analysis of intact mAbs. Therefore, to prevail over such hindrance (rapid transverse relaxation), different approaches were adopted, such as deuteration of non-labile ^1^H sites and utilization of the TROSY (transverse relaxation optimized spectroscopy) or CRINEPT (cross-correlated relaxation enhanced polarization transfer) [49,170]. Subsequently, an innovative ^15^N-direct revealing NMR analysis i.e., ^15^N-detected CRINEPT has also been reported that works on the same concept of coherence transfer as in the case of ^1^H-revealing CRINEPT and supports high-sensitive dimensions of non-deuterated high molecular weight proteins [75].

In this context, two principal 2D experiments for the 2D fingerprints having higher resolution than that of the 1D ^1^H NMR fingerprint were reported [66]. These included the ^1^H-^1^H NOESY (nuclear Overhauser effect spectroscopy) and the ^1^H-^13^C or ^1^H-^15^N HSQC (heteronuclear single quantum correlation) or HMQC (heteronuclear multiple quantum coherence) techniques. Moreover, 2D NMR also contributed towards acquiring the structural information of proteins’ fingerprints as well as their molecular interactions. It was also used for the determination of disulfide bond configurations and the characterization of PEGylation [74].

#### 4.3.3. Fourier Transform Infrared (FTIR) Spectroscopy

Fourier Transform Infrared Spectroscopy is an absorption spectroscopy that can be used to obtain information about the vibration bands (due to different functional groups, such as N-H, C=O). Furthermore, FTIR in tandem with CD spectroscopy has been reported for performing the overall structural characterization of the proteins [76,171]. The addition of glycan molecule at the particular sites within the amino acid chains of proteins represents the most common process of glycosylation (chief protein post-translational modification) and comprises about 60% of biopharmaceuticals mainly mAbs. The FTIR spectroscopy was reported recently to have application in comparing the glycosylation or monosaccharide profile in fourteen therapeutic mAbs samples (Adalimumab, Aflibercept, Bevacizumab, Cetuximab, Trastuzumab and emtasine) showing the spectral variations due to their compositions of glycan and monosaccharide [77,172]. Fourier-transform ion cyclotron resonance mass spectroscopy (FT-ICR-MS) was also introduced earlier to understand the structure/function relationship of proteins and their interactions [78]. In another report, the primary structure classification of mAbs (both N and C-terminal parts) was also analyzed through MALDI (Matrix-assisted laser desorption/ionization) in-source decay (ISD) MS [79,173]. Further, MALDI FT-ICR MS, was also utilized for monitoring the glycation levels of a bispecific monoclonal antibody (BsAb) (six different BsAb subunits namely two light chains, crystallizable and Fd regions) at subunit level [80].

#### 4.3.4. Ultraviolet-Visible (UV-Vis) Spectroscopy and Liquid Chromatography-Mass Spectrometry (LC-MS)

Ultraviolet-visible (UV-Vis) spectroscopy is an extensively used method in various fields of science starting from microbial culture, drug detection and nucleic acid quality assessment and estimation, to quality checking in the food industry or chemical research [174]. The UV-Vis spectroscopy is an analytical method that compares a sample to a reference or blank sample to see how many distinct wavelengths of UV or visible light are absorbed or transmitted. The sample composition has an impact on this characteristic, which might reveal information about sample constitution and concentration [175].

Several analytical methods, such as capillary electrophoresis (CE), LC-MS, HPLC, UV, IR or Raman spectroscopy and enzyme-linked immunosorbent assay (ELISA), used for the analyses of mAbs face demerits in terms of their time consumption, costlier instruments, and incompatible properties with saline solutions [81]. Therefore, two new apparatus applying UV/Raman spectroscopy and UV/IR namely QC prep+^®^ and Multispec^®^ respectively, were considered for the quality control of mAbs [176]. Jaccoulet et al. [81] reported a very simple and fast QC using FIA (Flow injection analysis) together with DAD (diode array detector) technique for the quantification of three important structurally similar mAbs, namely infliximab, rituximab, and bevacizumab from the hospital samples. Downstream analysis of mAbs is usually performed using chromatography; however, determining the concentration of co-eluting constituents poses a major problem. The ICM (Inline concentration measurements) through UV-Vis (Ultraviolet/Visible light) spectroscopic analysis offer a label-free and controlled method to considerably accelerate the mAbs testing and decrease the processing time [177].

Mass spectrometry (MS), such as LC-MS multi-attribute methods (MAM) demonstrated an imperative component in the structural characterization of mAbs. Moreover, to tackle the escalating analytical challenges of novel antibody formats, 2D-LC-MS methods emerged as key approaches during the last ten years [82,178,179]. Recently, the advantages and disadvantages of multidimensional-LC-MS approaches as compared to the other traditional chromatographic methods, i.e., 1D-LC-MS and 1D-LC-UV process at peptide and intact protein level, respectively, have been reviewed by Camperi et al. [82]. The progress of MS technology in biopharmaceutical industries has been gradually upgrading through execution of novel Ion Mobility mass spectroscopy (IMMS) for the identification of epitope and paratope of protein complexes [180]. Furthermore, the quantification of new humanized IgG_1_ therapeutic mAb against herpes simplex virus (HDIT101) was also reported to be carried out through the utilization of LC–MS-based bio-analytical tools [181].

The middle-up LC-MS technology for the intact mass measurement of mAbs (IgG_1_) and antibody drug conjugates (Random lysine conjugated) was earlier investigated by partially reducing the mAbs fragments by means of reduction and/or enzymatic cleavage [182]. Synthesis of mAbs involves several post-translational modifications (PTMs), specifically, glycosylation, which plays a critical role in the clinical efficiency and immunogenicity of therapeutic proteins [11]. In a recent study related to the development of modern drugs, LC–MS/MS-mediated glycoproteomic analysis was applied to two NISTmAb (RM 8671 and β-1,4-galactosidase-treated NISTmAb). There were no significant differences found in terms of glycan compositions in the two test samples [183,184]. The diverse analytical tools including MS for the characterization of commercially available Fc-fusion proteins have been elaborately illustrated by Duivelshof et al. [185]. Conclusively, the LC-MS technique became an integrative component and alternative spectroscopic tool for the structural characterization as well as quantification of important therapeutic drugs (mAbs) at the commercial level.

#### 4.3.5. Circular Dichroism (CD) Spectroscopy

Circular dichroism spectroscopy is an optical method that works on the principle of electronic transitions in the far ultraviolet (UV) wavelength region and is a widely utilized technique for determining the structures (HOS) and conformational changes of the mAbs in drug development [185,186,187]. During the last ten years, thousands of research papers have been published using this spectroscopic technique for the characterization of secondary and tertiary structures of diverse proteins [188]. This technique has several advantages over the other spectroscopic methods, such as (i) lesser sample quantity requirement [188], (ii) no harsh pre-treatment of the samples for revealing enantiomers, and (iii) execution of analysis in aqueous solutions [189]. Despite these aforesaid merits, the presently adopted method has certain limitations related to their weak signals for the spectroscopic measurements [190].

Sousa et al. [191] used CD spectroscopy to comprehend the alterations in the secondary and tertiary structures of the mAb-bevacizumab at various temperatures and pH. In yet another report, CD was employed for the characterization of a novel anti-HER2 mAb (5G4) along with Trastuzumab (Herceptin) as the reference standard [191,192]. Recently, another successful advancement in CD spectroscopic based analysis of mAbs was achieved through devising a website—DichroWeb (http://dichroweb.cryst.bbk.ac.uk/html/home.shtml; accessed on 22 August 2022) that included the reference datasets, ranges, and algorithms for determining the protein secondary structure (helix, sheet) from the spectroscopic data obtained from the popular tool circular dichroism [189,193].

### 4.4. Electrochemical Analyses

The mAbs are proving to be potent cancer therapeutic agents these days. Electrochemical analyses have been utilized for the characterization of the ligands of the mAbs, generally for the diagnostic purposes. Machini et al. [194] performed in situ characterization of novel mAb based anticancer drug, Nivolumab (NIVO)-dsDNA interaction employing DNA-Electrochemical Biosensor incorporating UV-Visible spectrophotometry, differential pulse voltammetry, gel electrophoresis, poly[A]- or poly[G]-electrochemical biosensors, electrochemical impedance spectroscopy, and quartz crystal microbalance. Quality assurance and the structure elucidation of the mAbs through top-down spectrometric methods remains complicated because of the protein size, disulfide bridges content, and post-translational modifications, such as glycosylation [195]. To resolve the complexity caused by disulfide linkages, Nicolardi et al. [174] took the help of electrochemical method for analyzing mAbs. In this approach, an electrochemical cell with a 15 T magnet was directly coupled to an ESI (electrospray ionization) source in addition to FTICR MS (Fourier transform ion cyclotron resonance mass spectrometer). This setup resulted in choosing the released light chains for tandem MS/MS analysis devoid of any intrusion due to heavy-chain fragments. The most prolific adducts and other interrupting species were identified using ultrahigh-resolution FTICR-MS observations, which offered fully resolved isotopic distributions of intact mAb enabling the detection of the most numerous adducts and other interfering species [174]. Wang et al. [196] created a label-free electrochemical sensor from murine mAb utilizing AuNPs (gold nanoparticles) and Ag-GO (silver-graphene oxide) nanocomposites to detect tiamulin (TML), which is a pleuromutilin antibiotic used in veterinary medicine for pigs and poultry. A broad detection range of TML between 0.01–1000 ng mL^−1^ was exhibited. Moreover, the effective and simple electrochemical immunosensor was used to accurately determine TML in real samples, indicating that it may be used to capture other veterinary antibiotics in animal-derived foods.

Various chromatographic methods, such as HPLC, GC-MS and ELISA, were earlier used for the determination of pesticides. However, their drawbacks regarding time consuming protocols, costlier instruments etc. led to the emergence of an alternative system, such as those using electrochemical sensors offering lower price, simplicity, and higher sensitivity for the detection of pesticides. The chiefly exploited global pesticide-Imidacloprid (IMD) was analyzed using specific mAb followed by SPCE (electrochemical detection on screen-printed carbon electrodes) that showed better performance than HPLC-MS and ELISA [83]. In an earlier study, a simple, cost-effective, and sensitive electrochemical immunoassay (immunosensor) method was developed for the determination of synthetic chemical colorants Sudan azo dyes Sudan I [197].

In the last few years, a lot of effort has been made into developing sophisticated electrochemical immunosensors, which selective analytical results by utilizing bio-recognition phenomenon between antigen and antibodies through application electrochemical transducer [198]. Recently, electrochemical ELISA-based immunosensor gained remarkable popularity and is advantageous because of the combination of optical ELISA with electrochemical methods. ELISA is useful for analysis of both antibodies as well as antigens [199]. These analytical devices have attracted the attention of researchers in the medical, pharmaceutical, and food industries due to benefits, such as high selectivity, susceptibility, portability, the ability to perform multi-analyte evaluation, low price, the ability to miniaturize, and smaller sampling requirements [199]. Despite this, it becomes imperative to enhance the attributes of electrochemical immunosensors by nurturing new ideas of antibody conjugation.

### 4.5. Recombinant DNA Technology

Another important technique used in the characterization of mAbs is the mRNA sequencing using recombinant DNA technology. The unique antigen binding domains present in each mAb is identified and its proper sequencing assists in functional evaluation and, therefore, the performance of rAbs [200]. This characterization is based on the sequencing of Ig transcripts achieved from mAb-producing hybirdomas followed by rAb expression. This sequencing can be combined with MS to achieve next-generation sequencing to characterize the VR sequences [200,201].

### 4.6. X-ray Diffraction Technique

The X-ray diffraction method is one of the widely used techniques to study the structure of proteins including mAbs along with understanding the protein-protein interactions. The antigen-antibody interaction, also called the epitope mapping, is widely elucidated in form of their complexes in crystals and characterized using X-ray diffraction [202]. The X-ray diffraction technique works on the principle of small-angle scattering (SAS) and provides low-resolution information about the shape of proteins. Several studies based on the SAS technique have been performed to determine the function, chemistry, and structure of mAbs [203,204,205], and were observed to be strongly complementing the conventional biophysical techniques.

Summarizing the major techniques discussed above, we can see that each method has its own advantage(s) and disadvantage(s) (Table 3). Many of them are compatible with mass spectrometry. Some are very costly and laborious, such as NMR spectroscopy, but it is the best for higher order structure elucidation of mAbs. Some techniques are cheap as well as allow high throughput and saves analysis time. Choosing the analytical method depends upon the user and the requirement for specific characterization of mAbs. Combinations of these techniques are applied for characterization by pharmaceutical industry to strictly comply the standards set by regulatory agencies.

## 5. Conclusions

In summary, detailed characterization of a biopharmaceutical product is a crucial part of drug development. An array of analytical techniques is required to characterize a monoclonal antibody fully and properly at different stages of its development and storage. These processes of characterization (physiochemical, structural, immunological, functional) must be strictly adhered to the guidelines of ICH and EMA to eventually release the biopharmaceutical drug in the market. The analytical tools discussed above have been used for characterization of mAbs related to their structure and stability, and to ensure their efficacy and safety. Chromatographic, electrophoretic, spectroscopic, and immunological techniques have individual merits and demerits. May times they are used in combination for desired and precise characterization of proteins. Analytical laboratories continually work towards improvements in their methodologies and instrumentation to obtain data having more precision, resolution, sensitivity, and reproducibility. Other advanced techniques further add merit towards the functional identification of mAbs in simple, shorter time frames and reliable form that will boost up the research and progress of forthcoming mAb therapeutics.

A plethora of chromatographic, electrophoretic, and spectroscopy methods and electrochemical analyses are available for the characterization of MABs. The methods described in the review provide a comprehensive coverage for mAbs characterization starting from RPLC, SEC, IEX to capillary electrophoretic, spectroscopic methods and the electrochemical analysis. For example, RPLC provides good resolution in protein variation evaluation due to some chemical process or post-translational modifications. On the other side RPLC-MS could be applied in separating antibody sub-domains while size exclusion chromatography helps in getting rid of heterogenic impurities during mAbs purification. The IEX chromatography is useful to separate the proteins of comparable sizes if they acquire different pI values. The main revolution has encountered in the recent years for tertiary structure determination of mAbs utilizing circular dichroism (CD), differential scanning calorimetry (DSC), proton-deuterium exchange mass spectrometry (HDX-MS), Raman and Fourier transform infrared spectroscopy (FTIR). However, each method or technique has its own uniqueness and limitation. Therefore, proper selection of the right method for specific characterization is desirable on the part or researcher. Characterization of mAb will continue to advance as next-generation technologies and methods are developed. Within the biopharma and biologics divisions, it will be a rising field for investigators.

## Figures and Tables

**Table 2:** Recent analytical techniques reported for the characterization of mAbs.

S. No.	Antibody Type	Analytical Technique	Reference
1.	Protease ClpP	^1^H-1D NMR, TROSY CRINEPT	[49]
2.	BrentuxiMAB vedotin	RPLC	[50]
3.	BevacizuMAB	RP-HPLC	[51]
4.	AdalimuMAB, atezolizuMAB, belimuMAB, bevacizuMAB, cetuxiMAB, dalatozuMAB, denosuMAB, eculizuMAB, elotuzuMAB, inflixiMAB, ipilimuMAB, ixekizuMAB, natalizuMAB, nivoluMAB, obinotuzuMAB, ofatumuMAB, panitumuMAB, pembrolizuMAB, pertuzuMAB, ramuciruMAB, reslizuMAB, rituxiMAB and trastuzuMAB	RPLC-MS	[52]
5.	Six different IgG1, one IgG2, one bispecific	RP-UPLC-MALS	[53]
6.	MAB oxidation variants	Mixed mode SEC	[54]
7.	MABs sourced from Genentech Inc., South San Francisco, CA	SE-UHPLC	[55]
8.	adalimuMAB, bevacizuMAB, cetuxiMAB, denosuMAB, natalizuMAB, ofatumuMAB palivizuMAB, panitumuMAB, rituxiMAB, trastuzuMAB	Cation exchange chromatography	[56]
9.	Different MABs with varying charges	Cation exchange chromatography-MS	[57]
10.	RituxiMAB, trastuzuMAB, bevacizuMAB, cetuxiMAB, inflixiMAB, and trastuzuMAB	Ion-Exchange Charge Variant Analysis	[58]
11.	InflixiMAB	CE-MS	[59]
12.	AdalimuMAB, atezolizuMAB, belimuMAB, bevacizuMAB, cetuxiMAB, denosuMAB, elotuzuMAB, ipilimuMAB, ixekizuMAB, nivoluMAB, obinituzuMAB, ofatumuMAB, palivizuMAB, pertuzuMAB, ramuciruMAB, rituxiMAB and trastuzuMAB	CZE	[60]
13.	TrastuzuMAB, inflixiMAB, ustekinuMAB	CE-MS	[61]
14.	MAB	CZE-MS	[62]
15.	RituxiMAB, trastuzuMAB and bevacizuMAB	CE-ESI-MS	[63]
16.	MAB	CZE	[64]
17.	NIST suggested MABs	imaged CIEF	[65]
18.	MABs	1D ^1^H NMR ^1^H-^1^H NOESY ^1^H-^13^C or ^1^H-^15^N HSQC or HMQC	[66]
19.	NISTMAB PS #8670	2D-NMR	[67]
20.	MAB	1D NMR with PROFOUND and NIPALS	[68]
21.	MABs	1D ^1^H NMR	[69]
22.	MABs	1D ^1^H NMR	[70]
23.	RituxiMAB and InflixiMAB	FPLC NMR, CD, SEC MALS	[71]
24.	Two IgG1 MABs (COE-03 and COE-19) and one bispecific IgG1MAB (COE-07)	1D ^1^H NMR	[72]
25.	MAB	1D ^1^H and DOSY NMR	[73]
26.	MAB	2D NMR	[74]
27.	MAB	2D-NMR with 15N-detected CRINEPT	[75]
28.	MAB	FTIR with CD	[76]
29.	AdalimuMAB, Aflibercept, BevacizuMAB, CetuxiMAB, InflixiMAB, NatalizuMAB, NivoluMAB, OmalizuMAB, PanitumuMAB, PembrolizuMAB, PertuzuMAB, RamuciruMAB, RituxiMAB, TrastuzuMAB, TrastuzuMAB-emtasine	FTIR	[77]
30.	MABs	FT-ICR-MS	[78]
31.	TrastuzuMAB NIST MAB	MALDI ISD MS	[79]
32.	Bispecific MAB	MALDI FT-ICR MS	[80]
33.	InflixiMAB, RituxiMAB, BevacizuMAB	FIA and DAD	[81]
34.	MABs	2D-LC-MS 1D-LC-MS 1D-LC-UV	[82]
35.	Anti-IMD (Imidacloprid), Antigen protein conjugate (BSA IMD)	SPCE (electrochemical detection on screen-printed carbon electrodes)	[83]
36.	CetuxiMAB	CZE-MS	[84]
37.	MABs	CZE	[85]
38.	IgG_1_ manufactured by Merck & Co., Inc. (Kenilworth, NJ, USA)	Two-Dimensional Liquid Chromatography (IEC-SEC 2D-LC)	[86]
39.	IgG_1_	RP-HPLC-MS	[87]
40.	TrastuzuMAB, rituxiMAB and palivizuMAB	CE-MS	[88]
41.	AdalimuMAB, natalizuMAB, nivoluMAB, palivizuMAB, inflixiMAB, rituxiMAB and trastuzuMAB	CE-MS	[89]
42.	FDA and EMA approved antibodies	SEC, IEX and hydrophobic interaction chromatography (HIC)	[90]
43.	MAB	2D-CZE-MS	[91]
44.	National Institute of Standards and Technology (NIST) MAB	LC-UV-MS	[92]
45.	Pfizer supplied MABs	CE	[93]
46.	InflixiMAB	CZE-MS	[94]
47.	IgG1	LC-MS	[95]
48.	MABs sourced from Genentech Inc., South San Francisco, CA	Multi-dimensional LC/MS	[96]

**Table 3:** Comparison of analytical methods used for antibody characterization with their respective advantages and disadvantages.

Methods		Basic Principle	Advantage(s)	Disadvantage(s)
Chromatographic	RPLC	Separation of the components based on their hydrophilicity/hydrophobicity	High resolving power and straightforward compatibility to mass spectrometry	RPLC-MS does not offer complete cover as small peptides are lost
	SEC	Common technique used to characterize size variants from biotherapeutic proteins	Good separation of large from small molecules;	Proteins with very comparable size cannot be resolved; any possibility of interaction between the stationary phase and the analyte leads to later elution leading to the inference of a smaller size analyte
	IEX	Very powerful technique to separate charged heterogeneity in biopharmaceuticals	It is used to remove impurities (protein aggregates, host cell proteins (HCPs), DNA and endotoxins); MS compatible	Column stability and reproducibility is not assured after repeated use
Electrophoretic	CZE	Popular separation technique based on the ratio of charge to mass of analytes	Sensitive, MS compatible and high throughput technique	Adsorption of mAbs on the capillary wall and requirement of method optimization
	CGE	Conventional separation method based on charge to mass ratio; utilizes gel in capillaries instead of liquid (as in CZE)	Allows analyses of product size heterogeneity, stability, and purity of mAbs.	MS and CGE-SDS are incompatible so SDS removal approaches have to be employed
	cIEF	Separation technique based on their isoelectric points	Analyzes charge heterogeneity, oxidation and deamidation analysis; MS compatible	Optimizing cIEF resolution and reproducibility can be challenging
Spectroscopic	1D ^1^H NMR	Based on nuclear magnetic resonance to elucidate HOS of mAbs	The most suitable method for determining the chemical shift and structural elucidation of the protein therapeutics	Requirements of isotope labelling, larger molecular weight and the restraints imposed due to numerous formulations have led to its decreased acceptability
	^1^H-based Multidimensional NMR	Higher resolution than 1D ^1^H NMR; Used for the identification of quality attributes (QA) and HOS characterization of mAbs	Detects precise atomic-level fingerprint of the primary/secondary/tertiary and quaternary structures of therapeutic mAbs	Lesser adaptability for structure analysis due to short transverse relaxation times along with the slow molecular tumbling, high cost of instrumentation
	FTIR	Absorption spectroscopy used to obtain information about the vibration bands (due to different functional groups such as N-H, C=O)	Non-destructive, high-resolution, fast technique	High cost and maintenance of instrument are undesirable features
	UV-Vis and LC-MS	Absorption/reflectance spectroscopy based on ultraviolet and visual spectrum	UV-Vis is a simple and convenient technique for protein quantification and aggregation. Compatible with LC-MS.	Not suitable in conditions where antibody or drug is susceptible to UV
	CD	Measures differences in the absorption of left- and right-handed circularly polarized light	Lesser sample quantity requirement, no harsh pre-treatment of the samples for revealing enantiomers and execution of analysis in aqueous solutions	Weak signals for the spectroscopic measurements
Electrochemical	Immunosensor	Based on specific antigen-antibody interaction followed by the conversion of the event to an electrical signal	Utilized for the characterization of the ligands of the mAbs, generally for the diagnostic purposes, portability, simple miniaturization, high sensitivity	Reproducibility; during the immunoreactions, changes in ion concentration, current, potential, and impedance can occur.

## Data Availability

Not applicable.
